# Supplementary material for: Content-rich biological network constructed by mining PubMed abstracts
Source: BMC Bioinformatics. 2004 Oct 8;5:147. doi: 10.1186/1471-2105-5-147 (PMC528731; doi:10.1186/1471-2105-5-147)
Supplement: Additional File 2 — The original results of the above study (non-essential files are deleted to keep the file size under the limit set by BMC bioinformatics). [file 1471-2105-5-147-S2.bz2 › chilibotAdditionalFile2/dip20/597ID8524402E10973/html/PPP3CA_PPP3R1.html]

 


 **PPP3CA** and **PPP3R1** 
  
Found 30 abstracts in PubMed, retrieved 20.  
 

 What does Google say? 
 PDF only 
| .edu only 

---

**Interactive relationship** (e.g. stimulation, inhibition, etc)

**Inhibitory relationship**- These results suggest a molecular mechanism for suppression of calcineurin by cyclosporin A and FK506 involving their binding to the same region of CnB  [ **PPP3R1** ]  used for allosterically activating CnA  [ **PPP3CA** ] .  Ref: 7525078 Cell, 1994
- The Ca2 dependent binding of phage displayed peptides to CnB  [ **PPP3R1** ]  and CaM is specifically blocked by synthetic peptides derived from the CnB  [ **PPP3R1** ]  binding domain of CnA  [ **PPP3CA** ]  and the CaM binding domain of myosin light chain kinase respectively.  Ref: 10072758 Gene, 1999
- CN is comprised of two subunits a 59 kDa catalytic subunit CNA  [ **PPP3CA** ] , which contains a calmodulin binding domain and autoinhibitory region, and a 19 kDa intrinsic calcium binding regulatory subunit CNB  [ **PPP3R1** ] .  Ref: 7523407 J Biol Chem, 1994
- The deduced amino acid sequence well conserves the calcineurin B CnB  [ **PPP3R1** ]  binding domain and two subdomains, a calmodulin binding and an autoinhibitory domain, showing 77 85% and 82% identities to the isoforms of Drosophila melanogaster CnA  [ **PPP3CA** ]  and human CnA  [ **PPP3CA** ] , respectively.  Ref: 11886782 Insect Biochem Mol Biol, 2002
**Neutral relationship**- Significantly, on a model of CnB  [ **PPP3R1** ] , these mutations map to the latch region, an element of tertiary structure that forms when CnB  [ **PPP3R1** ]  binds CnA  [ **PPP3CA** ] .  Ref: 7525078 Cell, 1994
- Its crystal structure reveals that the C terminal of CNB  [ **PPP3R1** ]  lies in close vicinity of the N terminal of CNA  [ **PPP3CA** ]  and each end has a long arm not involved in the active site.  Ref: 12890484 Biochem Biophys Res Commun, 2003
- Calcineurin B  **PPP3R1**  is located on human chromosome 2p16 >p15 and calcineurin A  [ **PPP3CA** ]  beta PPP3CB, previous gene symbol CALNB is present on 10q21 >q22.  Ref: 8978785 Cytogenet Cell Genet, 1996
- In contrast, we found that the regulatory CNB  [ **PPP3R1** ]  subunit appears to play an obligatory role in this interaction and have defined an amino acid sequence of the CNA  [ **PPP3CA** ]  subunit which forms the binding site for CNB  [ **PPP3R1** ] .  Ref: 7523407 J Biol Chem, 1994
- In kappa CnG, 148 and 168 codons coincide with the corresponding codons of kappa CnB  [ **PPP3R1** ] , and 136 and 167 codons correspond to kappa CnA  [ **PPP3CA** ] .  Ref: 9119217 Genetika, 1996
- ... their presence permits CnA1  [ **PPP3CA** ]  delta to bind more strongly to CnB  [ **PPP3R1** ] .  Ref: 7488022 Biochem Biophys Res Commun, 1995
- We have created calcineurins that are resistant to both cyclosporin A and FK506 by mutating specific residues in CnB  [ **PPP3R1** ] , a calcium binding protein that regulates the catalytic subunit, CnA  [ **PPP3CA** ] .  Ref: 7525078 Cell, 1994
- A yeast two hybrid system has demonstrated the molecular interaction between B. mori CnA  [ **PPP3CA** ]  and CnB  [ **PPP3R1** ] .  Ref: 11886782 Insect Biochem Mol Biol, 2002
- In vitro, both wild type and CNB1  [ **PPP3R1** ]  G2A mutant proteins formed complexes with both cyclophilin A cyclosporin A CsA and FKBP12 FK506 that contained calcineurin A  [ **PPP3CA** ] .  Ref: 7559604 J Biol Chem, 1995
- In order to examine this multicomponent system by dynamic NMR methods, the 93 kDa, tightly bound FKBP 12 FK506 Cn complex was replaced with a lower affinity, rapidly exchanging system consisting of FKBP 12 FK506 13 kDa, recombinant calcineurin subunit B CnB  [ **PPP3R1** ]  20 kDa, and a synthetic peptide 4 kDa corresponding to the B binding domain BBD of calcineurin catalytic subunit A CnA  [ **PPP3CA** ] .  Ref: 8880916 Protein Sci, 1996
- Immunoblot analysis indicated that increase in calcineurin activity was concordant with the increased expressions of calcineurin catalytic subunit isozymes, calcineurin A alpha  [ **PPP3CA** ]  CNA  [ **PPP3CA** ]  alpha, and calcineurin A  [ **PPP3CA** ]  beta CNA  [ **PPP3CA** ]  beta, and a regulatory calcineurin B subunit CNB  [ **PPP3R1** ]  proteins, which were preceded by a coordinate increase in the levels of CNA  [ **PPP3CA** ]  alpha, CNA  [ **PPP3CA** ]  beta and CNB  [ **PPP3R1** ]  mRNAs.  Ref: 8639915 Blood, 1996

**Non-interactive relationship** (e.g. studied together, co-existance, homology, etc.)

- Calcineurin CN is a Ca 2 calmodulin CaM dependent serine threonine protein phosphatase which is a heterodimer composed of a 61kDa catalytic subunit CNA  [ **PPP3CA** ]  and a 19kDa regulatory subunit CNB  [ **PPP3R1** ] .  Ref: 12890484 Biochem Biophys Res Commun, 2003
- The binding preferences identified with peptide libraries are consistent with the features of the CnB  [ **PPP3R1** ]  binding domains of all CnA  [ **PPP3CA** ]  isoforms and the CaM binding domains of CaM targets.  Ref: 10072758 Gene, 1999
- Here we report the identification and characterization of calcineurin genes in Caenorhabditis elegans cna 1  [ **PPP3CA** ]  and cnb 1  [ **PPP3R1** ] , which share high homology with Drosophila and mammalian calcineurin genes.  Ref: 12221132 Mol Biol Cell, 2002
- Calcineurin A alpha  [ **PPP3CA** ]   **PPP3CA** , calcineurin A  [ **PPP3CA** ]  beta PPP3CB and calcineurin B  **PPP3R1**  are located on human chromosomes 0q21 >q22 and 2p16 >p15 respectively.  Ref: 8978785 Cytogenet Cell Genet, 1996
- The use of several restriction endonucleases allowed three alleles of kappa casein kappa CnA  [ **PPP3CA** ] , kappa CnB  [ **PPP3R1** ] , kappa CnF to be typed and new allele variants in yak, European bison, and buffalo to be revealed.  Ref: 9119217 Genetika, 1996
- This suppression was found to be due to inhibition of interactions between activated calcineurin Cn and nuclear factor of activated T cells NF AT, inhibition of cleavage of the autoinhibitory domain of the CnA  [ **PPP3CA** ]  subunit, and inhibition of heterodimer formation by CnA  [ **PPP3CA** ]  and CnB  [ **PPP3R1** ]  subunits, resulting in the absence of NF AT in nuclei of the T cells.  Ref: 9182928 Arthritis Rheum, 1997
- Although deletion of the genes encoding the yeast catalytic subunits of calcineurin CNA1  [ **PPP3CA** ]  and CNA2 maintain the interaction, absence of the regulatory subunit Cnb1  [ **PPP3R1** ]  prevents binding.  Ref: 7528690 FEBS Lett, 1995
- The protein is a heterodimer of one catalytic CnA  [ **PPP3CA** ]  subunit and one regulatory CnB  [ **PPP3R1** ]  subunit.  Ref: 9199180 Biochem Biophys Res Commun, 1997
- Significantly, in the absence of its cognate cytosolic receptor, neither CsA nor FK506 inhibits or stimulates the CnA1  [ **PPP3CA** ]  delta AID, CnA1  [ **PPP3CA** ]  delta CnB  [ **PPP3R1** ]  interactions.  Ref: 7488022 Biochem Biophys Res Commun, 1995
- The results of Southern blotting experiments are compatible with the existence of multiple genes for CNA  [ **PPP3CA** ]  and a single gene for CNB  [ **PPP3R1** ] .  Ref: 11071287 Mol Biochem Parasitol, 2000
- In the Slovakian Spotted breed the frequencies of alleles were as follows kappa CnA  [ **PPP3CA** ]  = 0.666, kappa CnB  [ **PPP3R1** ]  = 0.333.  Ref: 7676573 Vet Med Praha , 1995
- Yeast cells lacking functional calcineurin cna1  [ **PPP3CA** ]  cna2 or cnb1  [ **PPP3R1** ]  mutants display growth defects under specific environmental conditions, for example, in the presence of high concentrations of Na, Li, Mn2, or OH but are indistinguishable from wild type cells under standard culture conditions.  Ref: 7542741 Mol Cell Biol, 1995
- Northern blot analyses revealed that both CnA  [ **PPP3CA** ]  and CnB  [ **PPP3R1** ]  genes were expressed in various larval and adult tissues of B. mori.  Ref: 11886782 Insect Biochem Mol Biol, 2002
